# High adherence to all-oral directly acting antiviral HCV therapy among an inner-city patient population in a phase 2a study

**DOI:** 10.1007/s12072-015-9680-7

**Published:** 2015-11-26

**Authors:** Tess Petersen, Kerry Townsend, Lori A. Gordon, Sreetha Sidharthan, Rachel Silk, Amy Nelson, Chloe Gross, Monica Calderón, Michael Proschan, Anu Osinusi, Michael A. Polis, Henry Masur, Shyam Kottilil, Anita Kohli

**Affiliations:** Critical Care Medicine Department, National Institutes of Health Clinical Center, National Institutes of Health, Bethesda, MD USA; Laboratory of Immunoregulation, National Institute of Allergy and Infectious Diseases, National Institutes of Health, Bethesda, MD USA; Clinical Center Pharmacy Department, National Institutes of Health, Bethesda, MD USA; Xavier University of Louisiana, New Orleans, LA USA; Division of Infectious Diseases, Institute of Human Virology, University of Maryland, Baltimore, MD USA; Clinical Research Directorate/Clinical Monitoring Research Program, Leidos Biomedical Research, Inc. (formerly SAIC–Frederick, Inc.), Frederick National Laboratory for Cancer Research, Frederick, MD USA; Food and Drug Administration, Silver Spring, MD USA; Biostatistics Research Branch, NIAID, NIH, Bethesda, MD USA; Gilead Sciences Inc., Foster City, CA USA; Division of Hepatology, St. Josephs Hospital and Medical Center, Creighton University Medical School, Phoenix, AZ USA; Division of Clinical Care and Research, Institute of Human Virology, N222, University of Maryland School of Medicine, 725 West Lombard St, Room S222, Baltimore, MD 21201 USA

**Keywords:** HCV, DAA, Adherence, MEMS

## Abstract

**Background:**

As treatment for chronic hepatitis C (HCV) virus has evolved to all-oral, interferon-free directly acting antiviral (DAA) therapy, the impact of these improvements on patient adherence has not been described.

**Methods:**

Medication adherence was measured in 60 HCV, genotype-1, treatment-naïve participants enrolled in a phase 2a clinical trial at the National Institutes of Health and community clinics. Participants received either ledipasvir/sofosbuvir (LDV/SOF) (90 mg/400 mg) (one pill) daily for 12 weeks, LDV/SOF + GS-9451 (80 mg/day) (two pills) daily for 6 weeks, or LDV/SOF + GS-9669 (500 mg twice daily; three pills, two in the morning, one in the evening) for 6 weeks. Adherence was measured using medication event monitoring system (MEMS) caps, pill counts and patient report.

**Results:**

Overall adherence to DAAs was high. Adherence declined over the course of the 12-week treatment (*p* = 0.04). While controlled psychiatric disease or symptoms of depression did not influence adherence, recent drug use was a risk factor for non-adherence to 12-week (*p* = 0.01), but not 6-week regimens. Adherence as measured by MEMS was lower than by patient report.

**Conclusions:**

Adherence to short courses of DAA therapy with 1–3 pills a day was excellent in an urban population with multiple risk factors for non-adherence.

**Electronic supplementary material:**

The online version of this article (doi:10.1007/s12072-015-9680-7) contains supplementary material, which is available to authorized users.

## Introduction

Chronic hepatitis C virus (HCV) affects over 180 million people worldwide and results in approximately 300,000 deaths annually due to cirrhosis and 200,000 deaths due to hepatocellular cancer [1]. Treatment for HCV has rapidly evolved—from complex regimens associated with multiple toxicities that require weekly injections of pegylated-interferon (IFN) in combination with eight pills a day for 12–48 weeks—to new regimens of all-oral directly acting antiviral (DAA) agents only characterized by low pill burdens, short therapeutic courses, few side effects, and high (>90 %) rates of sustained virologic response (SVR) [2–9]. Most recently, the DAA-only regimen consisting of a fixed-dose combination of ledipasvir and sofosbuvir (LDV/SOF) was approved by the Food and Drug Administration (FDA) for the treatment of chronic HCV genotype 1 (GT-1) infection [9].

Evaluating adherence to DAA-only regimens is vital to translating the high efficacy of these regimens observed in phase III clinical trials to the community. In particular, adherence is important to attaining the maximal rate of SVR from a treatment regimen, avoiding treatment failure and/or the development of DAA resistance [7, 8]. Additionally, the cost of medication non-adherence across all diseases has been estimated to exceed $100 billion in additional healthcare expenditures [6]. Given the high cost of HCV DAA medications, which has driven some insurers to implement rules authorizing only one course of DAA therapy in a patient’s lifetime, maximizing SVR rates is especially important [10, 11].

While low adherence (<80 %) to IFN-containing regimens without DAAs has been shown to result in decreased rates of SVR, the impact of patient adherence on outcomes in DAA-only therapy has not been established [12]. As RNA virus, HCV rapidly replicates and is highly error prone, suggesting that it could develop high-level resistance to DAAs [7, 13]. The subsequent risk of emerging class-specific viral mutants at treatment failure may restrict future treatment options [14]. Therefore, understanding the impact of duration, pill burden, and patient psychosocial factors on adherence to DAA-only regimens is critical to developing simple, efficacious treatments vital to achieving global eradication of HCV.

To evaluate the potential impact of patient adherence to HCV treatment on outcomes, the adherence of patients enrolled in three arms of the NIH SYNERGY trial was measured.

## Patients and methods

### Study design and patients

**Fig. 1:**
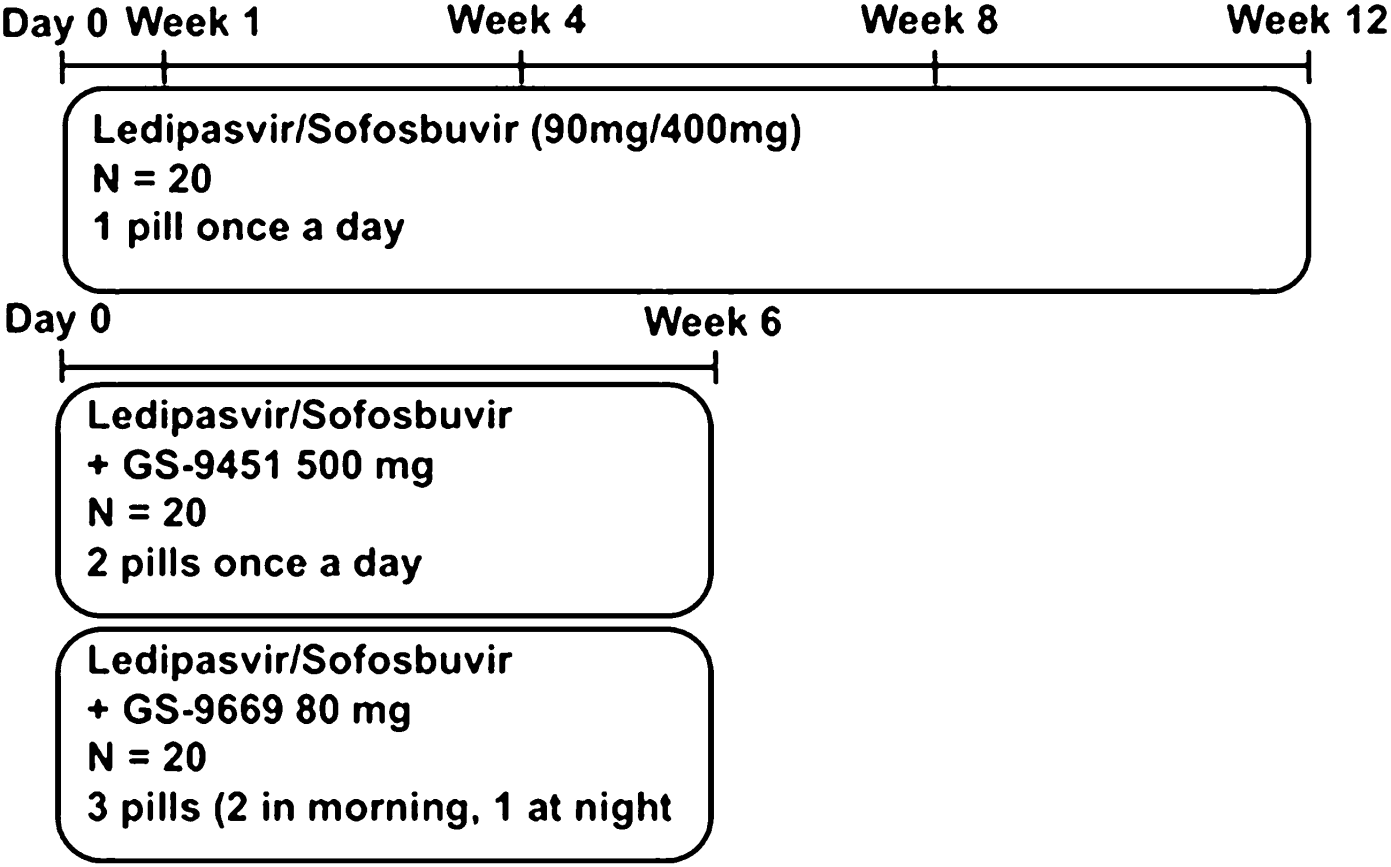
Study design and adherence visits

The SYNERGY study was designed to investigate whether the addition of a third potent DAA to LDV/SOF enables treatment duration to be shortened from 12 to 6 weeks, while maintaining high efficacy. Sixty HCV mono-infected, treatment-naïve, GT-1 patients were enrolled into one of three arms in this phase 2a clinical trial and received: LDV/SOF (90 mg/400 mg) one pill once daily for 12 weeks (*n* = 20), LDV/SOF + GS-9451 (80 mg/day) two pills once daily for 6 weeks (*n* = 20), or LDV/SOF + GS-9669 (500 mg/day) three pills (two of LDV/SOF and GS-9669 in the morning and one of GS-9669 in the evening) for 6 weeks (*n* = 20) (Fig. 1). Full inclusion and exclusion criteria have been previously published [15]. Written or oral informed consent approved by the National Institute of Allergy and Infectious Diseases (NIAID) Institutional Review Board (IRB) was obtained from all participants.

### Community model: DC partnership for AIDS/HIV progress

The DC Partnership For AIDS Progress (DC-PFAP) is a collaborative initiative funded by the Office of AIDS Research to improve the care of HIV-infected subjects in the District of Columbia in collaboration with the DC Department of Health and the National Institutes of Health (NIH). As part of this program, a hepatitis subspecialty team treats HCV at multiple clinics established within community health centers in Washington, DC. All screening and day 0 (study medication initiation) visits took place at the NIH Clinical Center. Subsequent study visits were performed at the community subspecialty clinics or at the NIH based on subject preference. Patients referred through DC-PFAP belonged to an inner-city population reflective of the US HCV epidemic.

### Main outcome measures

Primary outcome in this substudy was adherence as measured by three tools: medication event monitoring system (MEMS) caps, pill counts, and patient reports. We aimed to describe adherence to novel DAA-only treatment regimens and to investigate the risk factors associated with non-adherence to these regimens.

### Adherence measures

Adherence visits occurred at day 0 (start of study drug) and end of treatment for all patients. Patients treated with the 12-week regimen had additional adherence visits at weeks 1, 4, and 8 (Fig. 1). During day 0 adherence visits, patients were provided seven standardized points describing the adherence tools (Supplemental Table 1) and were assured that non-adherence would not affect study participation. In order to maintain optimal levels of study medications in their blood, patients were counseled to take the study medications at approximately the same time every day. A dose taken correctly was measured within a time frame of 24 ± 2 h from the previous dose, except in the case of GS-9669, which was to be taken 12 h ± 2 h from the previous dose. Patients were instructed to bring their medication bottles to each study visit so that the study team could perform a pill count and collect MEMS data. Results of adherence tools were collected by clinical trial staff and entered into an electronic database. Results were reviewed weekly by the principal investigator, but, with one exception, were not discussed with patients.

**Table 1:** Demographics and baseline characteristics

	Ledipasvir/sofosbuvir (*n* = 20)	Ledipasvir/sofosbuvir + GS-9451 (*n* = 20)	Ledipasvir/sofosbuvir + GS-9669 (*n* = 20)	*p* value
Age (years)	57 ± 8	54 ± 9	54 ± 7	0.28
Male	14 (70)	16 (80)	13 (65)	0.56
Black race*	16 (80)	18 (90)	19 (95)	0.32
History of psychiatric diagnosis	*n* = 11	*n* = 10	*n* = 13	
Bipolar D/o	3 (27)	1 (10)	2 (15)	0.62
Depression	4 (37)	6 (60)	6 (46)	
Schizophrenia	1 (9)	1 (10)	0 (0)	
Other	3 (27)	2 (20)	5 (39)	
Alcohol consumption last 30 days (Y)	1 (5)	2 (10)	3 (15)	0.37
Marijuana last 6 months (Y)	6 (30)	2 (10)	6 (30)	0.48
Cocaine last 6 months (Y)	2 (10)	1 (5)	2 (10)	0.96
Heroin last 6 months (Y)	0 (0)	1 (5)	2 (10)	0.40
Highest education > 12th grade	9 (45)	6 (30)	7 (35)	0.17
Work for pay outside the home (Y)	9 (45)	3 (18)	4 (22)	0.14
Self-reported risk for HCV				
Shared drug paraphernalia	9 (45)	10 (50)	11 (55)	0.99
Blood transfusion	3 (15)	1 (5)	0 (0)	
Shared drug paraphernalia + blood transfusion	2 (10)	3 (15)	4 (20)	
Don’t know/not reported	14 (70)	16 (80)	14 (70)	

Data are mean (SD) or *n* (%)

*HCV* hepatitis C virus

* Race was self-reported

### Medication event-monitoring systems (MEMS)

Electronic MEMSCaps (MWV Switzerland Ltd., Sion, Switzerland) were placed on study medication bottles at day 0. Patients treated with LDV/SOF for 12 weeks had medication refills at weeks 4 and 8, and MEMSCaps were transferred to the new pill bottles by study team members at these time points. Patients treated on the 6-week regimens of LDV/SOF and GS-9451 or GS-9669 received all 6 weeks of medication at day 0. For patients taking multiple pills, color-coded MEMSCaps were placed on color-coded bottles to help patients more easily match the correct cap to its bottle. Dosing history (including date and time of dose and missed doses) was blindly extracted from MEMSCaps [Advanced Analytical Research on Drug Exposure Group (AARDEX), Sion, Switzerland] at each adherence visit.

### Pill count

The number of pills remaining in each patient's bottles as well as the number of pills dispensed were recorded at each adherence visit and were used to calculate the total number of missed doses between adherence visits.

### Patient report

The study team adapted questionnaires previously developed and validated by the AIDS Clinical Trials Group (ACTG), which were widely used for measuring adherence and risk factors for non-adherence to HIV antiviral drugs [16]. Questionnaires assessed three main areas: (1) adherence self-efficacy and beliefs about medication effectiveness, (2) psychological distress and social support including questions from the Center for Epidemiological Studies Depression (CES-D) scale (total of seven four-point items) and the Perceived Stress Scale (total of four five-point items), and (3) alcohol and drug use [16]. Baseline and follow-up ACTG questionnaires were modified for this study to be applicable for patients infected with HCV alone (Supplemental Text 1–4). Baseline questionnaires were administered at day 0 and follow-up questionnaires at subsequent adherence visits. Clinical trial staff was available to read questionnaires to patients with low literacy and were available to patients with questions.

### Statistical analysis

Adherence across and within treatment arms was compared using ANOVA and *t* tests. Means and standard errors are reported. Risk factors for non-adherence were assessed using chi-squared tests, *t* tests, or Pearson’s correlations as appropriate. Multivariate analyses were not performed, given the small sample size.

## Results

Demographics and patient characteristics are shown in Table 1. Eighty-seven percent of study participants were recruited from HCV clinics associated with the DC-PFAP program. While some patients chose to remain at the NIH Clinical Center, the majority of patients (57 %) transitioned back to their community clinics at or after week 4.

Most patients were male (72 %), African American (88 %), had a high-school degree or less (63 %), and had a diagnosed psychiatric disease (57 %). Ten percent, 17, 8, and 5 % of patients had abused alcohol, marijuana, cocaine, and/or heroin, respectively, in the 6 months prior to starting the study medications. Twenty-seven percent of patients were employed outside of their home. Intravenous drug use (IVDU) was the most common self-reported risk factor for HCV (52 %), followed by IVDU along with blood transfusions (15 %) and blood transfusions alone (7 %). Twenty-eight percent of patients did not answer or did not know their risk factors for HCV infection.

### High adherence to medications by MEMS, pill counts, and patient report

**Fig. 2:**
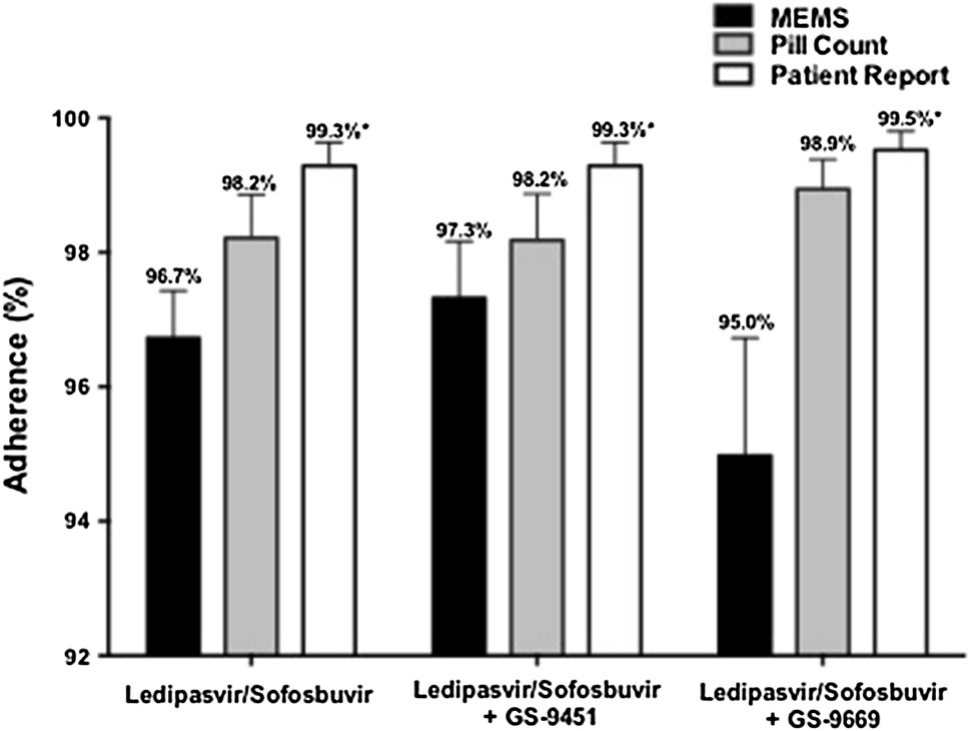
Adherence to DAA regimens measured by MEMS, pill count, and patient report. *p < 0.05 versus MEMS

Adherence to DAAs was high as measured by MEMS, pill count, and patient report. In patients treated with 12 weeks of LDV/SOF in a once daily combination tablet, the overall adherence was 97.6, 98.2, and 99.3 % by MEMS, pill count, and patient report, respectively. In patients treated with LDV/SOF and GS-9451 for 6 weeks with two pills once daily, overall adherence was 97.3, 98.2, and 99.3 % by MEMS, pill count, and patient report, respectively. In the regimen using LDV/SOF and GS-9669 with three pills (two in the morning, one in the evening) daily, overall adherence was 95.0, 98.9, and 99.5 % by MEMS, pill count, and patient report, respectively (Fig. 2). There was no difference in overall adherence among the three regimens by MEMS (*p* = 0.36), pill count (*p* = 0.60), or patient report (*p* = 0.84).

Within each regimen, adherence as measured by MEMS was consistently lower than that measured by patient self-report (LDV/SOF, *p* = 0.002; LDV/SOF + GS-9451, *p* = 0.01; LDV/SOF + GS-9669, *p* = 0.01). Conversely, adherence as measured by MEMS was similar to that reported by pill count for the one-pill and two-pill per day treatment arms (LDV/SOF, *p* = 0.13; LDV/SOF + GS-9451, *p* = 0.28), but was significantly lower than that by pill count for patients receiving the three pill per day regimen of LDV/SOF and GS-9669 (*p* = 0.04). There was no significant difference in adherence as measured by pill count and patient report (LDV/SOF, *p* = 0.15; LDV/SOF + GS-9451, *p* = 0.15; LDV/SOF + GS-9669, *p* = 0.26) (Fig. 2).

Self-reported reasons for missed doses by MEMS or pill count are summarized in Table 2. The most common reasons included, “feeling as if the treatment was working” (38 %), “forgetting” (35 %), and “being away from home” (32 %).

**Table 2:** Self reported reasons for non-adherence among non-adherent patients by MEMS or pill count

Self-reported reasons for missed doses *n* (%)	Ledipasvir/sofosbuvir	Ledipasvir/sofosbuvir + GS-9451	Ledipasvir/sofosbuvir + GS-9669	Total
	*n* = 14	*n* = 9	*n* = 14	*n* = 37
Felt like treatment was working	8 (57)	2 (22)	4 (28)	14 (38)
Simply forgot	7 (50)	4 (44)	2 (14)	13 (35)
Away from home	6 (42)	5 (56)	1 (7)	12 (32)
Had a change in daily routine	4 (28)	2 (22)	2 (14)	8 (22)
Felt depressed/overwhelmed	6 (43)	1 (11)	0 (0)	7 (19)
Felt worse when took medication	5 (35)	0 (0)	2 (14)	7 (19)
Felt sick/ill	3 (21)	3 (33)	1 (7)	7 (19)
Fell asleep/slept through dose time	4 (28)	1 (11)	1 (7)	6 (16)
Felt like drug was harmful or toxic	3 (21)	1 (11)	0 (0)	4 (11)
Had problems taking at specified times	3 (21)	0 (0)	1 (7)	4 (11)
Felt hassled or inconvenienced by medicine	2 (14)	1 (11)	0 (0)	3 (8)
Too many pills to take	2 (14)	1 (11)	0 (0)	3 (8)
Did not want others to notice	2 (14)	1 (11)	0 (0)	3 (8)
Ran out of pills	1 (7)	0 (0)	1 (7)	2 (5)

Adherence results were not discussed with patients in order to mimic a “real-world” clinic experience where MEMS data would not be routinely available. One exception was a single patient who by MEMS was found to have missed five doses by the week 4 visit. Given that the patient had advanced liver disease with cirrhosis, the principal investigator decided that the risk of not counseling the patient outweighed any benefit to the adherence study. The patient was counseled by a study physician to improve adherence but subsequently missed six additional doses over the next 8 weeks.

### Pill burden and adherence to DAAs

**Fig. 3:**
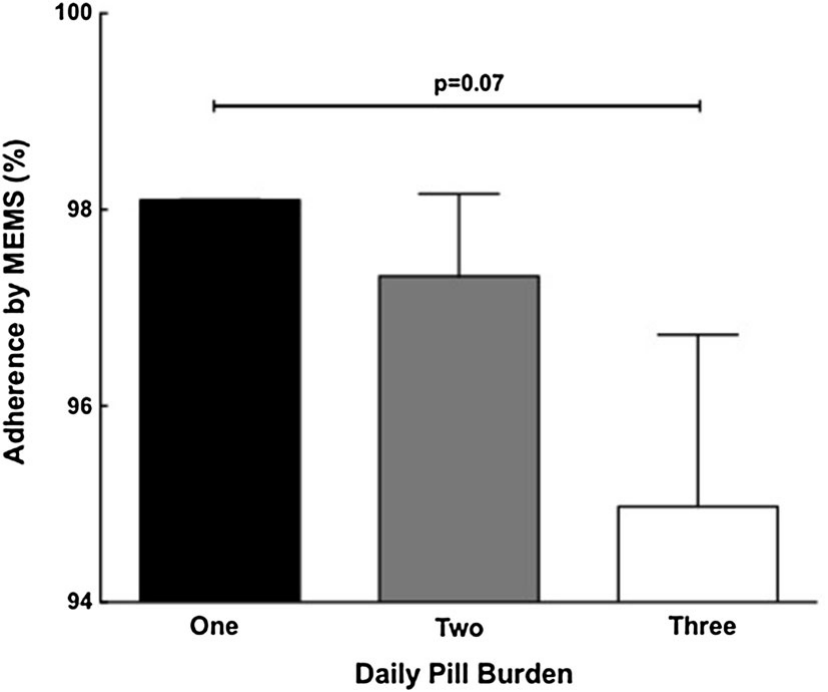
Adherence to DAAs decreases with increasing pill burden. Adherence to DAA regimens declined with increasing pill burden (*first 6 weeks compared between arms)

In order to assess the impact of pill burden on adherence, adherence during the initial 6 weeks of therapy was compared across treatment arms. A trend toward lower adherence was observed in the LDV/SOF + GS-9669 three pill per day regimen (two in the morning, one at night) as compared to the LDV/SOF one pill per day regimen (1 pill/day: 98.2 ± 0.02 % vs. 3 pills/day: 95.0 ± 1.75 %; *p* = 0.07) (Fig. 3).

### Adherence to DAAs declines with increasing treatment duration

**Fig. 4:**
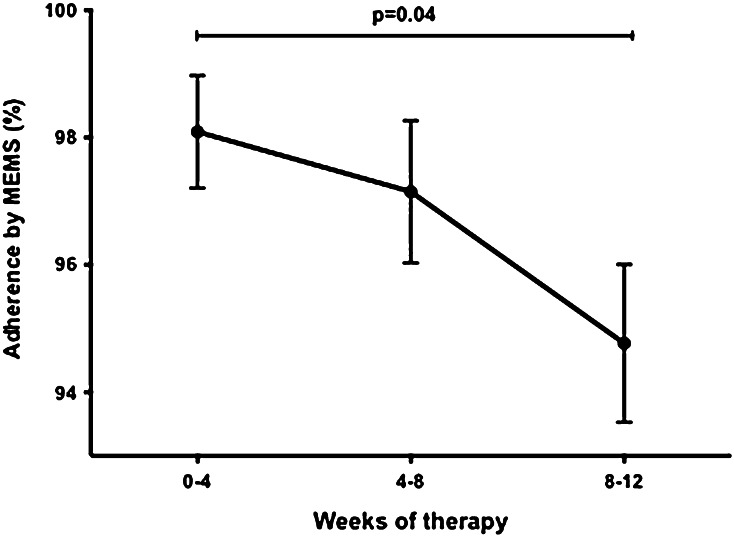
Adherence to DAAs declines over 12-week treatment course. Adherence between weeks 0–4, 98.1 ± 0.9 %, was significantly higher than adherence between weeks 8–12, 95.0 ± 1.2 %. for patients treated with 12 weeks of LDV/SOF

To control for the influence of pill burden while determining the impact of treatment duration on adherence, adherence of participants over the course of the 12-week regimen of LDV/SOF was compared. Adherence to this regimen as measured by MEMS declined over the course of therapy (week 0–4: 98.1 ± 0.9 % vs. week 8–12: 95.0 ± 1.2 %; *p* = 0.04) (Fig. 4).

### Demographic risk factors for non-adherence to directly acting antiviral therapy

In a univariate analysis of demographic and baseline characteristics, mean adherence did not differ based on psychiatric disease, symptoms of depression (i.e., CES-D score ≥ 8), perceived stress level (PSS), gender, education, work status, children, or patient perceived self-efficacy (Table 3) [17, 18].

**Table 3:** Risk factors for patient non-adherence to directly acting antivirals for treatment of hepatitis C

	Ledipasvir/sofosbuvir	*p* value	Ledipasvir/sofosbuvir + GS-9451	*p* value	Ledipasvir/sofosbuvir + GS-9669	*p* value
Sex**: **mean adherence ± SD (*n*)						
Male	96.7 ± 0.9 (14)	0.93	96.8 ± 1.1 (14)	0.73	95.2 ± 2.6 (10)	0.87
Female	96.8 ± 1.1 (6)		97.6 ± 0.0 (3)		94.6 ± 2.5 (8)	
Age*	*R* ^2^ = 0.02	0.59	*R* ^2^ = 0.025	0.54	*R* ^2^ = 0.019	0.59
Race						
White	99.1 ± 0.9 (4)	0.09	96.4 ± 3.6 (2)	0.85	97.6 (1)	N/A**
Black	96.1 ± 0.8 (16)		97.0 ± 0.9 (15)		94.8 ± 1.9 (17)	
Highest education: mean adherence ± SD (*n*)						
Masters	100.0 (1)	0.34***	None	0.36	None	0.88
College graduate (4 year)	100.0 (2)		95.2 ± 0.0 (1)		92.9 ± 4.8 (3)	
College graduate (2 year)	95.2 ± 1.5 (6)		99.4 ± 1.2 (4)		98.8 + 1.2 (2)	
High school graduate	96.6 ± 0.9 (9)		96.6 ± 1.6 (7)		94.4 ± 3.6 (8)	
11th grade or less	97.02 ± 3.0 (2)		95.7 ± 1.9 (5)		95.2 ± 1.7 (4)	
Currently employed						
Yes	97.8 ± 0.8 (9)	0.19	98.4 ± 1.6 (3)	0.73	98.2 ± 1.1 (4)	0.28
No	95.9 ± 1.1 (11)		97.6 ± 1.1 (11)		93.3 ± 2.5 (12)	
Recent substance abuse						
Yes	96.4 ± 1.4 (8)	0.01	98.4 ± 0.8	0.19	92.5 ± 3.8 (7)	0.28
No	99.4 ± 0.3 (12)		95.6 ± 1.8 (6)		96.5 ± 1.5 (11)	
Diagnosed psychiatric disease						
Yes	95.8 ± 1.5 (7)	0.32	97.3 ± 1.3 (7)	0.75	92.6 ± 3.4 (8)	0.23
No	97.3 ± 0.7 (13)		96.7 ± 1.3 (10)		96.9 ± 1.6 (10)	
Center for Epidemiological Studies depression (CES-D) score^#^						
CES-D ≥8			97.8 ± 0.6 (9)			0.40
CES-D <8			96.2 ± 0.85 (43)			
Self-reported self-efficacy						
You will be able to take all or most of the study medication as directed?						
Not at all/somewhat/very sure	96.0 ± 1.9 (6)	0.53	95.7 ± 1.9 (5)	0.41	89.3 ± 6.5 (4)	0.08
Extremely sure	97.0 ± 0.6 (14)		97.4 ± 1.0 (12)		96.6 ± 1.2 (14)	
The medication will have a positive impact on your health?						
Not at all/somewhat/very sure	96.7 ± 1.1 (11)	0.73	96.6 ± 1.3 (9)	0.47	94.8 ± 2.5 (11)	0.91
Extremely sure	97.2 ± 0.9 (8)		98.0 ± 1.3 (7)		95.2 ± 2.3 (7)	
In general, how satisfied are you with the overall support you get from your friends and family members?						
Very or somewhat dissatisfied/somewhat satisfied	96.8 ± 1.7 (3)	0.95	97.2 ± 1.6 (6)	0.81	89.3 ± 6.5 (4)	0.08
Very satisfied	96.7 ± 0.79 (17)		96.8 ± 1.2 (11)		96.6 ± 1.2 (14)	
To what extent do your friends or family members help you remember to take your medication?						
Not at all/A little/somewhat	96.6 ± 0.8 (8)	0.47	98.6 ± 1.0 (5)	0.53	95.2 ± 2.3 (7)	0.82
A lot	96.2 ± 1.3 (9)		96.3 ± 1.5 (9)		95.9 ± 2.2 (7)	
Not applicable	98.8 ± 1.2 (3)		96.0 ± 2.1 (3)		92.9 ± 6.3 (4)	
Do you have any children?						
Yes	96.3 ± 0.8 (15)	0.27	97.4 ± 1.2 (10)	0.92	92.9 ± 1.7 (12)	0.61
No	98.1 ± 1.2 (5)		97.1 ± 1.9 (5)		91.1 ± 3.1 (4)	
Do any children live with you?						
Yes	97.4 ± 0.7 (15)	0.09	97.9 ± 1.1 (9)	0.90	95.9 ± 1.9 (11)	0.30
No	94.4 ± 2.3 (4)		97.6 ± 1.8 (5)		91.4 ± 4.7 (5)	
Perceived stress score*	*R* ^2^ = 0.0003	0.94	*R* ^2^ = 0.14	0.14	*R* ^2^ = 0.029	0.50

* Pearson’s correlations

** Cannot compare with one value *n* = 1

*** Excluding master's degree, which only had 1 value

^#^CES-D Score ≥8 associated with depression. Unable to perform analysis for each arm given the small numbers of patients with CES-D score ≥8

In the 12-week treatment arm, adherence to LDV/SOF was significantly lower among participants who used drugs (including marijuana, cocaine, or heroin) in the 6 months prior to starting DAA therapy or abused alcohol (>3 drinks per day or >5 drinks in a 2–4-h period at any time during the 30 days) (*p* = 0.01) (Table 3, defined as “Recent substance abuse”). However, in the 6-week treatment arms, patients with a recent history of alcohol abuse and/or drug use had similar adherence to the DAA regimen as patients without this history (LDV/SOF + GS-9451, *p* = 0.19; LDV/SOF + GS-9669, *p* = 0.28).

### Timing of DAA administration and consecutive missed doses

In the LDV/SOF treatment arm, patients took 72 ± 18 % of doses at the correct time. In the LDV/SOF and GS-9451 or GS-9669 arms, patients took 64 ± 18 and 61 ± 18 % of doses at the correct time, respectively. There was no difference in the mean number of doses taken at the correct time among the three regimens (LDV/SOF vs. LDV/SOF + GS-9451, *p* = 0.16; LDV/SOF vs. LDV/SOF + GS-9669, *p* = 0.08; LDV/SOF + GS-9451 vs. LDV/SOF + GS-9669, *p* = 0.70).

Given that missing doses of DAA therapy may increase the risk of developing HCV resistance-associated variants (RAV) or enrich populations of existing RAVs, we examined the relationship between the number of consecutive missed doses by MEMS and enrichment of baseline mutations [14]. Only 4 out of 60 patients missed two or more consecutive doses of study medications by MEMS. One patient treated with LDV/SOF for 12 weeks and one patient treated with LDV/SOF + GS-9669 missed two consecutive doses. There were two additional patients on the three-pill regimen that missed consecutive doses: one missed three, four, and four consecutive doses, and one missed five consecutive doses. All of these patients achieved SVR.

### Correlation of missed doses with SVR

Fifty-eight (96.6 %) of 60 patients in the study achieved SVR. One patient relapsed 2 weeks after completion of therapy. This patient missed one dose by both MEMS and pill count and zero doses by patient report. The other patient was incarcerated after achieving SVR4, and no further data are available.

## Discussion

In this phase 2a study, adherence to all-oral DAAs for HCV treatment was high among a largely inner-city patient population with multiple risk factors for treatment noncompliance. Although adherence declined with increases in pill burden and treatment duration, mean adherence as measured by MEMS did not fall below 95 % irrespective of these factors. Recent drug use was associated with decreased treatment compliance in the 12-week arm, but not in the 6-week arms. Additionally, neither concomitant controlled mental illness nor symptoms of anxiety or depression influenced adherence. Due to the efficacy of DAA regimens and the high adherence observed in this study, no risk factors could be associated with viral relapse. Overall, simple, short-duration, all-oral HCV therapy in patients with perceived risk factors for treatment noncompliance may be as efficacious as in populations without these risk factors.

The development of DAAs has dramatically simplified treatment for hepatitis C by improving cure rates and decreasing side effects [2–4, 19]. The real-world effectiveness of all-oral, interferon- and ribavirin-free DAA regimens is similar to that seen in clinical trials [20, 21]. Given that missed doses of HCV treatment may result in the potential development of viral resistance, adherence to these DAA medications remains an important issue as larger numbers of HCV-infected patients are treated [7, 14, 22].

Participants in this study were generally representative of the patient population infected with HCV in the USA and demonstrated sociodemographic characteristics associated with medication non-adherence [23–25]. Although patients with active drug use, active alcohol abuse, or uncontrolled psychiatric disease were excluded from the study, diagnosed psychiatric disease was common, and many patients reported recent drug use. Data from this study show that medication adherence in patients with stable psychiatric disease or symptoms of depression (as measured by CES-D) is similar to that in patients without psychiatric disease [26]. Recent drug use was associated with decreased adherence in patients treated with the longer 12-week treatment duration, suggesting that shorter, simple regimens may be better for this patient population. These regimens may be administered in the setting of drug rehabilitation for patients not at high risk of HCV reinfection, where additional monitoring and adherence support is available.

Adherence as measured by the MEMS and pill count was similar between the one-pill (LDV/SOF) and two-pill (LDV/SOF + GS-9451) per day regimes, but a trend toward lower adherence was observed in the three-pill (LDV/SOF + GS-9669) per day (two pills in the morning, one pill at night) regimen. It is unknown whether the additional pill burden or the additional time of pill administration had a greater bearing on this decreased adherence. It should be noted that for all three regimens, adherence as measured by MEMS was lower than that determined by patient report, but similar to that by pill count. This supports the finding that when prompted to report adherence rates, patients tend to overestimate their adherence and underestimate their number of missed doses [27]. Given the similar measurements obtained by MEMS and pill count and the high cost of MEMS caps, clinicians can consider pill count an alternative and reliable measure for monitoring patient adherence.

While forgetting their medications and being away from home were among the top reasons for patients missing HCV medications, the most common reason, as reported by 38 % of patients, was feeling as if the medications were working. During this study, patients would meet with a member of the study team at each visit and discuss their progress on study. Anecdotally, many patients were interested and happy to see their rapid HCV viral load decline upon initiation of therapy. Patients were counseled that these results, while promising, did not indicate SVR. Given that many patients reported their reason for non-adherence as feeling reassured that the medications were working, additional counseling about the meaning of HCV viral load decline may be needed to improve adherence, particularly in non-study settings where patient interactions with their provider may be more limited.

Limitations of this study include the small sample size, which restricts the ability to perform a multivariate analysis of risk factors for non-adherence. Since drug and alcohol abuse was self-reported by the patients, there may be an underestimation of these characteristics among our patient population. Additionally, adherence as studied here may be different from real-world clinical practice. Patients in this study were enrolled on a clinical trial to test novel DAA therapies for the treatment of HCV and thus had frequent study visits with their healthcare provider, which may serve to reinforce adherence to medications. However, only one subject who missed 11 of 84 doses had an intervention to address poor adherence. The remaining patients were observed, which may be comparable to a clinical setting where adherence by MEMS caps and pill counts are not routinely performed. The results of this study warrant future evaluations of adherence in patients undergoing DAA therapy in real-life settings worldwide, specifically for those DAA-based therapies that extend over 12 weeks and include ribavirin, which is still part of the standard of care for HCV in most parts of the world. Given the high degree of adherence and high percentage of SVR in this study, we were not able to demonstrate the threshold of adherence required to achieve SVR. This is an important clinical question that needs to be addressed in future clinical trials with a large number of patients as DAA therapy becomes more widely used globally.

Although several studies of adherence have addressed issues related to long-term chronic illnesses, there are few studies investigating adherence to shorter medical regimens of 12–24 weeks or less of therapy [28, 29]. In this study, we found that during a one-pill once-daily 12-week regimen, adherence to medication dropped off after 4 weeks, and significantly after 8 weeks. Therefore, efforts to devise DAA regimens for HCV treatment of no longer than 8 weeks should be encouraged.

In conclusion, adherence to DAAs was high in this urban population of patients who are representative of the HCV epidemic in the US [23]. Numerous potent regimens are in development for the treatment of HCV, thereby providing clinicians with multiple options for their patients [3, 4, 15, 19, 30]. When selecting the optimal treatment, clinicians should consider pill burden and treatment duration in addition to cost, adverse events, efficacy, and drug-drug interactions. Additionally, clinicians should be aware that HCV treatment of patients with controlled psychiatric disease or symptoms of depression may be equally successful as that in patients without psychiatric disease.

## Electronic supplementary material

Supplementary material 1 (PDF 405 kb)
