# Supplementary material for: High adherence to all-oral directly acting antiviral HCV therapy among an inner-city patient population in a phase 2a study
Source: Hepatol Int. 2015 Nov 26;10(2):310–9. doi: 10.1007/s12072-015-9680-7 (PMC4778154; doi:10.1007/s12072-015-9680-7)
Supplement: Supplementary file 1 — Supplementary material 1 (PDF 405 kb) [file 12072_2015_9680_MOESM1_ESM.pdf]

SUPPLEMENTARY APPENDIX

Supplement to: Tess Petersen, Kerry Townsend, et al. High Adherence to All Oral Directly Acting Antiviral HCV Therapy Among Inner City Patient Population in Phase 2A Study

Contents

Supplemental Table 1: Seven Points Covered At Initiation of Directly Acting Antiviral Therapy for Hepatitis C **page 2**

Supplemental Text 1: Questionnaire used at baseline (modified form AACTG Questionnaire) **page 3**

Supplemental Text 2: Questionnaire used in follow up for 12 weeks of Ledipasvir/Sofosbuvir at Day 7, week 4, week 8, and week 12 (modified form AACTG Questionnaire)
Referred to as "Arm A" on the Questionnaire **page 9**

Supplemental Text 3: Questionnaire used in follow up for 6 weeks of Ledipasvir/Sofosbuvir + GS-9451 at week 6 (modified form AACTG Questionnaire)
Referred to as "Arm C" on the Questionnaire **page 11**

Supplemental Text 4: Questionnaire used in follow up for 6 weeks of Ledipasvir/Sofosbuvir + GS-9669 at week 6 (modified form AACTG Questionnaire)
Referred to as "Arm B" on the Questionnaire **page 13**

Supplemental Table 1: Seven Points Covered At Initiation of Directly Acting Antiviral Therapy for Hepatitis C

- Adherence measures were in order to correlate study outcomes with adherence after completion of study, and as such, would not affect participation/continuation in the study at any point
- Instruction regarding proper use of MEMS cap: Patients are to only open MEMS cap when ready to take dose and close MEMS cap following dose
- Patients instructed not to use a pill box for study drug
- Patient is to take her/his dose at exactly the same time each day
- Appropriate dosing of study drug reviewed with patients (1 tablet PO once daily (arm A), 2 tablets PO once daily (arm C), or 3 tablets PO once daily (arm B) with or without food
- Patients advised that it is OK to take dose (if forgotten), when she/he remembers within same day. Patients advised to not to double any doses (if dose forgotten until the next day)
- Patients reminded to bring Rx bottle to every visit

Adherence Baseline Questionnaire

		Self	Interviewer	Both
Date: ______________________				
Patient ID: ______________________	How Administered?	❑	❑	❑
Study Visit: ______________________				

The answers you give on this form will be used to plan ways to help other people who must take pills on a difficult schedule. Please do the best you can to answer all the questions. If you do not wish to answer a question, please draw a line through it. If you do not know how to answer a question, ask your study nurse to help. Thank you for helping in this important study.

INSTRUCTIONS: Please answer the following questions by placing a circle around the appropriate number response.

A. How sure are you that:

Please circle one response for each question.

	Not at All Sure	Somewhat Sure	Very Sure	Extremely Sure
1. You will be able to take all or most of the study medication as directed?	0	1	2	3
2. The medication will have a positive effect on your health?	0	1	2	3

B. The following questions ask about your social support.

Please circle one response for each question.

	Very Dissatisfied	Somewhat Dissatisfied	Somewhat Satisfied	Very Satisfied
1. In general, how satisfied are you with the overall support you get from your friends and family members?	0	1	2	3

	Not At All	A Little	Somewhat	A Lot	Not Applicable
2. To what extent do your friends or family members help you remember to take your medication?	0	1	2	3	4

C. People may miss taking their medications for various reasons. Here is a list of possible reasons why you may have missed taking any medications within the **past month**.

If you have **NOT** taken **any** medications within the **past month**, please check this box and skip to Section **E**. ☐

In the past month, how often have you missed taking your medications because you:

Please circle one response for each question.

		Never	Rarely	Sometimes	Often
1.	Were away from home?	0	1	2	3
2.	Felt hassled or inconvenienced by medicine?	0	1	2	3
3.	Simply forgot?	0	1	2	3
4.	Had too many pills to take?	0	1	2	3
5.	Felt worse when you took pills?	0	1	2	3
6.	Did not want others to notice you taking medication?	0	1	2	3
7.	Had a change in daily routine?	0	1	2	3
8.	Felt like the drug was toxic/harmful?	0	1	2	3
9.	Fell asleep/slept through dose time?	0	1	2	3
10.	Felt sick or ill?	0	1	2	3
11.	Felt depressed/overwhelmed?	0	1	2	3
12.	Had problem taking pills at specified times (with meals, on empty stomach, etc.)?	0	1	2	3
13.	Ran out of pills?	0	1	2	3
14.	Felt like treatment was working?	0	1	2	3

D. When was the last time you missed taking any of your medications? Check one box.

- ☐ Within the past **week**
- ☐ 1-2 **weeks** ago
- ☐ 2-4 **weeks** ago
- ☐ 1-3 **months** ago
- ☐ More than 3 **months** ago
- ☐ **Never** skip medications or **not applicable**

E. In the past week how often did you:

Please circle one response for each question.

		Never/ Rarely	Sometimes	Often	Mostly or Always
1.	Feel like you couldn't shake off the blues even with help from your family or friends?	0	1	2	3
2.	Have trouble keeping your mind on what you were doing?	0	1	2	3
3.	Feel that everything you did was an effort?	0	1	2	3
4.	Have trouble sleeping?	0	1	2	3
5.	Feel lonely?	0	1	2	3
6.	Feel sad?	0	1	2	3
7.	Feel like you just couldn't "get going"?	0	1	2	3

F. In the past month, how often have you:

Please circle one response for each question.

		Never	Almost Never	Sometimes	Fairly Often	Very Often
1.	Been upset because of something that happened unexpectedly?	0	1	2	3	4
2.	Felt unable to control the important things in in your life?	0	1	2	3	4
3.	Felt nervous and "stressed"?	0	1	2	3	4
4.	Felt confident in your ability to handle your personal problems?	0	1	2	3	4
5.	Felt that things were going your way?	0	1	2	3	4
6.	Found that you could not cope with all the things that you had to do?	0	1	2	3	4
7.	Been able to control irritations in your life?	0	1	2	3	4
8.	Felt that you were on top of things?	0	1	2	3	4
9.	Been angered because of things that happened that were outside of your control?	0	1	2	3	4
10.	Felt problems were piling up so high that you could not overcome them?	0	1	2	3	4

G. People have various health habits. The following questions ask about your alcohol and drug use, past and current.

1. How often have you had a drink containing alcohol – a glass of beer, wine, a mixed drink, or any kind of alcoholic beverage – in the last 30 days? Check one.

Daily	Nearly Every Day	3 or 4 Times A Week	Once or Twice A Week	2 or 3 Times A Month	Once A Month	Never
❑	❑	❑	❑	❑	❑	❑

*If **Never**, skip ahead to question **#4**.*

2. On days when you drank any alcoholic beverages in the last 30 days, how many drinks did you usually have altogether? By a drink we mean a can or glass of beer, a 4-ounce glass of wine, a 1-1/2 ounce shot of liquor, or a mixed drink with 1-1/2 ounces of liquor? Check one.

1 or 2 Drinks Per Day	3 or 4 Drinks Per Day	5 or 6 Drinks Per Day	7 or 8 Drinks Per Day	9 - 11 Drinks Per Day	12 or more Drinks Per Day
❑	❑	❑	❑	❑	❑

3. During the past 30 days, how often have you had 5 or more drinks of alcohol in a row, that is, within a couple of hours (e.g. 2-4 hours)? Check one.

Daily	Nearly Every Day	3 or 4 Times A Week	Once or Twice A Week	2 or 3 Times A Month	Once A Month	Never
❑	❑	❑	❑	❑	❑	❑

4. Please check "Yes" or "No" for each question.

a. ❑ Yes ❑ No **Have you ever used marijuana?**
If you used this drug, have you used it within the past 6 months?

❑ Yes ❑ No

b. ❑ Yes ❑ No **Have you ever used cocaine (powder, crack, or freebase)?**
If you used this drug, have you used it within the past 6 months?

❑ Yes ❑ No

c. ❑ Yes ❑ No **Have you ever used heroin?**
If you used this drug, have you used it within the past 6 months?

❑ Yes ❑ No

d. ❑ Yes ❑ No **Have you ever used amphetamines (speed)?**
If you used this drug, have you used it within the past 6 months?

❑ Yes ❑ No

H. These last questions ask about your background.

1. What is the highest level of education you have completed? (check one)

- ❑ 11th grade or less
- ❑ High school graduate or GED
- ❑ 2 years of college / AA degree / Technical school training
- ❑ College graduate (BA or BS)
- ❑ Master's Degree
- ❑ Doctorate / medical degree / law degree

2. What is (are) the most likely way(s) that you became infected with hepatitis C? (check "Yes" or "No" for each question.)

a. Shared needles with a person who was Hepatitis C+

❑ Yes ❑ No

b. Shared straws (snorting) with a person who was Hepatitis C+

❑ Yes ❑ No

c. Blood transfusion or other medical procedure

❑ Yes ❑ No

d. Sex with a person who was Hepatitis C+

❑ Yes ❑ No

e. Don't know

❑ Yes ❑ No

f. Other (needle stick at work, etc.)

❑ Yes ❑ No

Please specify: ______________________________

3. Do you work for pay outside the home? ❑ Yes ❑ No

4. Do you have any children? ❑ Yes ❑ No

If Yes, how many live with you? ______

Adherence Questionnaire: GS-7977/5885 FDC Arm

Date ___________________

Study ID ___________________

Study Visit ___________________

	Self	Interviewer	Both
How Administered?	❑	❑	❑

INSTRUCTIONS:

The answers you give on this form will be used to plan ways to help other people who must take pills on a difficult schedule. Please do your best to answer all of the questions honestly. We need to know what is really happening, so don't worry about telling us that you don't take your pills. If you do not wish to answer a question, please draw a line through it. If you do not know how to answer a question, ask a member of the study team to help. Thank you for helping in this important study.

1. Please complete the following table by filling in the boxes below.

	HOW MANY DOSES DID YOU **MISS** . . .			
**Step 1**<br>Hepatitis C study drug:	**Step 2**<br>Yesterday	**Step 3**<br>Day before yesterday (2 days ago)	**Step 4**<br>3 days ago	**Step 5**<br>4 days ago
GS-7977/5885: 1 pill, 1 dose per day				

2. Some people find that they forget to take their pills on the weekend days. Did you miss any of your hepatitis C medications last weekend– last Saturday or Sunday?

❑ Yes ❑ No

3. When was the last time you missed any of your medications? Check one.

❑ Within the past **week**

❑ 1-2 **weeks** ago

❑ 2-4 **weeks** ago

❑ 1-3 **months** ago

❑ **Never** skip medications or **not applicable**

4. People may miss taking their medications for various reasons. Here is a list of possible reasons why you may miss taking your medications. How often have you missed taking your medications because you: (Circle one response for each question.)

	Never	Rarely	Sometimes	Often
1. Were away from home?	0	1	2	3
2. Felt hassled or inconvenienced by medicine?	0	1	2	3
3. Simply forgot?	0	1	2	3
4. Had too many pills to take?	0	1	2	3
5. Felt worse when you took pills?	0	1	2	3
6. Did not want others to notice you taking medication?	0	1	2	3
7. Had a change in daily routine?	0	1	2	3
8. Felt like the drug was toxic/harmful?	0	1	2	3
9. Fell asleep/slept through dose time?	0	1	2	3
10. Felt sick or ill?	0	1	2	3
11. Felt depressed/overwhelmed?	0	1	2	3
12. Had problems taking pills at specified times (with meals, on empty stomach, etc.)?	0	1	2	3
13. Ran out of pills?	0	1	2	3
14. Felt like treatment was working?	0	1	2	3

SYNERGY ADHERENCE QUESTIONNAIRE: GROUP C

Date ________________ Study ID ________________ Study Visit ________________

1. How many doses of GS-7977/GS-5885 (blue pill) have you missed since your last study visit? ___________

2. How many doses of GS-9451 have you missed since your last study visit? ___________

3. Since your last study visit, how often did you take your study medications with food? Check one.

- ❑ All of the time
- ❑ Most of the time
- ❑ About half of the time
- ❑ Some of the time
- ❑ Never

4. Some people find that they forget to take their pills on the weekend days. Did you miss any of your hepatitis C medications last weekend– last Saturday or Sunday?

❑ Yes ❑ No

5. When was the last time you missed any of your study medications? Check one.

- ❑ Within the past week
- ❑ 1-2 weeks ago
- ❑ 2-4 weeks ago
- ❑ 1-3 months ago
- ❑ Never skip medications or not applicable

6. People may miss taking their medications for various reasons. Here is a list of possible reasons why you may miss taking your medications. Have you ever missed taking your study medications because you: (Circle one response for each question.)

		Never	Rarely	Sometimes	Often
1.	Were away from home?	0	1	2	3
2.	Felt hassled or inconvenienced by medicine?	0	1	2	3
3.	Simply forgot?	0	1	2	3
4.	Had too many pills to take?	0	1	2	3
5.	Felt worse when you took pills?	0	1	2	3

SYNERGY ADHERENCE QUESTIONNAIRE: GROUP C

6.	Did not want others to notice you taking medication?	0	1	2	3
7.	Had a change in daily routine?	0	1	2	3
8.	Felt like the drug was toxic/harmful?	0	1	2	3
9.	Fell asleep/slept through dose time?	0	1	2	3
10.	Felt sick or ill?	0	1	2	3
11.	Felt depressed/overwhelmed?	0	1	2	3
12.	Had problems taking pills at specified times (with meals, on empty stomach, etc.)?	0	1	2	3
13.	Ran out of pills?	0	1	2	3
14.	Felt like treatment was working?	0	1	2	3

SYNERGY ADHERENCE QUESTIONNAIRE: GROUP B

Date ________________ Study ID ________________ Study Visit ________________

1. How many doses of GS-7977/GS-5885 (blue pill) have you missed since your last study visit? ___________

2. How many doses of GS-9669 (white pills) have you missed since your last study visit? ___________

3. Since your last study visit, how often did you take your study medications with food? Check one.

- ❑ All of the time
- ❑ Most of the time
- ❑ About half of the time
- ❑ Some of the time
- ❑ Never

4. Some people find that they forget to take their pills on the weekend days. Did you miss any of your hepatitis C medications last weekend– last Saturday or Sunday?

❑ Yes ❑ No

5. When was the last time you missed any of your study medications? Check one.

- ❑ Within the past week
- ❑ 1-2 weeks ago
- ❑ 2-4 weeks ago
- ❑ 1-3 months ago
- ❑ Never skip medications or not applicable

6. People may miss taking their medications for various reasons. Here is a list of possible reasons why you may miss taking your medications. Have you ever missed taking your study medications because you: (Circle one response for each question.)

		Never	Rarely	Sometimes	Often
1.	Were away from home?	0	1	2	3
2.	Felt hassled or inconvenienced by medicine?	0	1	2	3
3.	Simply forgot?	0	1	2	3
4.	Had too many pills to take?	0	1	2	3
5.	Felt worse when you took pills?	0	1	2	3

SYNERGY ADHERENCE QUESTIONNAIRE: GROUP B

6.	Did not want others to notice you taking medication?	0	1	2	3
7.	Had a change in daily routine?	0	1	2	3
8.	Felt like the drug was toxic/harmful?	0	1	2	3
9.	Fell asleep/slept through dose time?	0	1	2	3
10.	Felt sick or ill?	0	1	2	3
11.	Felt depressed/overwhelmed?	0	1	2	3
12.	Had problems taking pills at specified times (with meals, on empty stomach, etc.)?	0	1	2	3
13.	Ran out of pills?	0	1	2	3
14.	Felt like treatment was working?	0	1	2	3
